# The Effects of Erbium-Doped Yttrium Aluminum Garnet Laser (Er: YAG) Irradiation on Sandblasted and Acid-Etched (SLA) Titanium, an In Vitro Study

**DOI:** 10.3390/ma13184174

**Published:** 2020-09-19

**Authors:** Antonio Scarano, Felice Lorusso, Francesco Inchingolo, Francesca Postiglione, Morena Petrini

**Affiliations:** 1 Department of Medical, Oral and Biotechnological Sciences, University of Chieti-Pescara, 66100 Chieti, Italy; francesca-postiglione@hotmail.com (F.P.); morena.petrini@unich.it (M.P.); 2 Zirconia Implant Research Group (Z.I.R.G), International Academy of Ceramic Implantology, Silver Spring, MD 20910, USA; 3 Department of Oral Implantology, Dental Research Division, College Ingà, UNINGÁ, Cachoeiro de Itapemirim 29312, Brazil; 4 Department of Medical, Oral and Biotechnological Sciences and CeSi Met, University of Chieti-Pescara, 66100 Chieti, Italy; drlorussofelice@gmail.com; 5 Department of Interdisciplinary Medicine, University of Bari “Aldo Moro,” 70121 Bari, Italy; f.inchingolo@icloud.com

**Keywords:** laser, Er: YAG, peri-implantitis, roughness, biofilm, SLA, dental implants

## Abstract

The treatment of peri-implantitis implies the decontamination of the surface of the fixture. This study aims to analyze the effect of the erbium-doped yttrium aluminum garnet laser (Er: YAG) on sandblasted and acid-etched (SLA) titanium. 30 titanium SLA disks were divided into three groups. In Group 1, the disks were left intact; on the contrary, both Groups 2 and 3 were irradiated with the Er: YAG laser at different settings, with a pulse duration of 300 μs and a period of 30 s. Group 2 was irradiated at 1 W and 100 mJ/pulse and Group 3 at 4 W and 400 mJ/pulse. The superficial changes at chemical, nano, and microscopical levels were detected through the use of Fourier-transform infrared spectroscopy, atomic force microscopy, and scanning electron microscope. The Kruskal–Wallis test, followed by the Dunn–Bonferroni Post Hoc analysis, detected the presence of statistically significant differences among the groups. The level of significance was *p* ≤ 0.05. Results showed that Er: YAG irradiation promoted a significant (*p* < 0.05) increase of oxides and a decrease of microscopical roughness and porosity on SLA disks. However, the protocol tested on group 3 seemed to be too aggressive for the titanium surface, as shown by the presence of micro-cracks and signs of coagulation, melting, and microfractures. In conclusion, Group 2 showed significantly minor surface alterations with respect to Group 3, and the increase of superficial oxide level, the decrease of porosity, and micro-roughness represent a positive alteration that could protect the materials against bacterial adhesion.

## 1. Introduction

Dental implants are widely used for the rehabilitation of partially or fully edentulous patients. Today, one in four patients rehabilitated with the implant-supported prosthesis are likely to undergo a peri-implant disease at some point in their life [1]. Peri-mucositis is a pathological reversible condition characterized by the inflammation in the peri-implant tissues, but when the process is associated with a progressive loss of supporting bone, it is defined as peri-implantitis [2]. The etiological factors of peri-implant disease are partly similar to those of periodontal disease: the high presence of biofilm around the implants is the predisposing factor for the development of peri-implant pathology [3,4].

The recognized risk indicators of peri-implantitis include the history of severe periodontitis, poor plaque control, and lack of periodic dental check-ups. Still unclear, however, is the evidence of the favoring role of smoking and diabetes on the onset of the disease. Finally, little literature allows us to draw conclusions on the role of keratinized gingiva, occlusal overload, bone tissue compression, its overheating and necrosis, micro-movements, the presence of titanium particles in peri-implant tissues, and bio-corrosion [5].

Currently, the detoxifying and decontamination of the implant surface, with the reduction of peri-implant pockets, represent the treatment of choice, and it could provide the restoration of supportive bone tissues and the re-osseointegration [6]. However, the process of implant decontamination and biofilm inactivation could represent a challenging treatment, especially in the case of treated surfaces that increase the superficial roughness [6,7]. Antibiotics fail to penetrate biofilm, and their clinical use is limited for peri-implantitis; on the other hand, mechanical methods could alter the implant surface, increasing the risk of inflammation in the surrounding tissues for the release of metallic particles [5,8].

In recent years, due to the increasing phenomenon of antibiotic resistance, the literature supports the use of red and near-infrared (NIR) light-based devices as an additive or alternative methods to provide an antibacterial and anti-biofilm effect [9,10,11,12,13]. In particular, the use of the Erbium-Doped Yttrium Aluminum Garnet Laser (Er: YAG) has been sustained as an additive or alternative instrument for dental implants’ decontamination in cases of peri-implantitis, thanks to its known anti-calculus and antibacterial activity [14,15,16,17].

An in vitro study of Tosun et al. has shown that 10 s of Er: YAG irradiation at 90 mJ and 10 Hz of frequency in a super pulse mode of 300 ms achieved 100% of Staphylococcus aureus elimination on sandblasted, large-grit, acid-etched surface titanium discs [18].

The Er: YAG emitting light at a wavelength of 2.94 μm is characterized by a great affinity for water, so its use is versatile on both soft and hard tissues [19]. Considering that the reflection capacity of titanium (Ti) for the Er: YAG light is 71%, implant surfaces should not absorb the irradiation, and subsequently, the temperature should not increase during the decontamination processes, and no damages to the implant surface should occur [20,21]. However, titanium in contact with air quickly adsorbs and reacts with other elements, like carbon, oxygen, and nitrogen, forming an amorphous oxide layer (TiO_2_) [22].

Consequently, there is a contrast in the literature, for what concerns the possible effects of Er: YAG laser irradiation on titanium surfaces: some studies have reported no thermal effects or detectable surface alterations [14,16,21,23], others have shown the presence of superficial alterations, like, melting, coagulation, and microfractures [24,25,26,27]. The reason is due to factors related to the different implant surfaces and to the various parameters used for laser decontamination. Different implant surfaces interact in a different manner to Er: YAG treatments. Kreiser et al. have shown that using a 0.5 mm distance from the tip, without water cooling, the energy necessary to induce surface alterations was: 8.9, 11.2, 17.8, and 28.0 Jcm^−2^ on the titanium plasma spray (TPS), sandblasting and acid etching (SLA), hydroxyapatite-coated (HA), and machine-polished surfaces, respectively [27]. The resultant interaction between the Er: YAG laser irradiation and implant surfaces affected both the topography and the composition of the superficial layer [24]. Another study showed that the bactericidal effect of Er: YAG irradiation is dependent on bacterial strain, laser settings, and the type of titanium surface: 10 s of Er: YAG irradiation at 500 mJ, 10 Hz of frequency, and 250–400 us were effective in reducing Streptococcus sanguinis below the detection limit on SLA but not on polished titanium [15]. The same authors also found superficial titanium alterations due to the laser treatment.

The aim of this study was to evaluate the effects at the chemical, nano and microscopical level of different powers and durations of Er: YAG laser irradiation on SLA titanium disks in order to find the better clinical protocol.

## 2. Materials and Methods

### 2.1. Sample Size Calculation

The sample size was determined based on the study of Kim et al. [28] that calculated a minimum of 3 samples for each group through a preliminary study. However, considering that we had to perform 3 different analyses for each group, Atomic Force Microscopy (AFM), Fourier-transform infrared spectroscopy (FTIR), and Scanning Electron Microscope (SEM), a minimum of 9 disks for each group were necessary.

### 2.2. Titanium Disks

**Figure 1:**
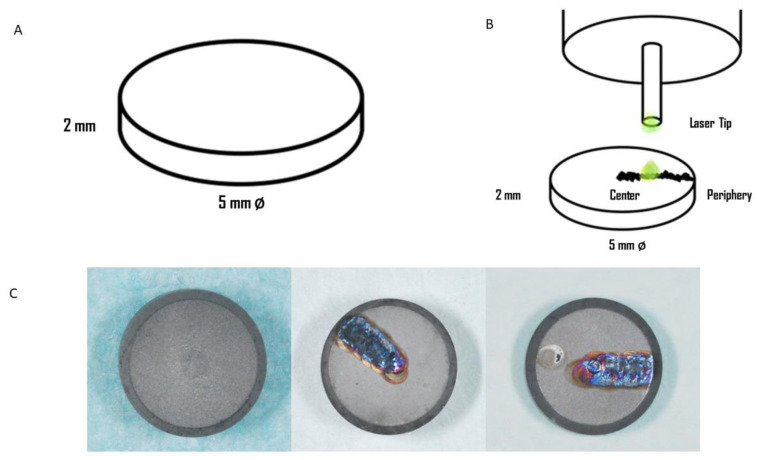
(**A**) Disks used in the present study. (**B**) The titanium disks during laser irradiation. (**C**) The sandblasted and acid-etched surface before and after the laser irradiation.

Commercially pure titanium (Isomed, Padova, Italy) disks of 5 mm in diameter and 2 mm in thickness with sandblasted and acid-etched surface were used (Figure 1A). All the samples were fully packed and sterilized, and the packages were opened just before laser irradiation. In total, 30 disks of titanium grade 4 were used for this research, divided into 3 groups:

- Group 1: 10 disks without any treatments: controls
- Group 2: 10 disks subjected to Er: YAG laser treatment at 1 W and 100 mJ/pulse
- Group 3: 10 disks subjected to Er: YAG laser treatment at 4 W and 400 mJ/pulse

### 2.3. Laser Device

An Er: YAG laser (Fidelis, Fotona, Ljubljana, Slovenia) with a wavelength of 2940 nm and a sapphire tip has been used in this study. The pulse frequency of 10 Hz and pulse durations up to 300 μs for a period of 30 s were chosen as recommended by the manufacturer.

The laser was applied perpendicular to the disk with a beam that focused on a spot size of 0.4 mm at 1 mm from the application tip. The water flow rate was set manually at 8 mL/min, and the tip was moved with sweeping movements. The laser tip moved from the periphery to the center of the disk in parallel movements with constant velocity (Figure 1B,C). After laser irradiation, all thirty disks were collected in a sterile and clean container.

### 2.4. Surface Analyses

The surface characterization of the disks was performed using Atomic Force Microscopy (AFM), Fourier-transform infrared spectroscopy (FTIR), and Scanning Electron Microscope (SEM).

#### 2.4.1. Atomic Force Microscopy

The AFM, NX10 Park AFM instrument (Park System, Suwon, Korea) was equipped with 20-bit closed-loop XY and Z flexure scanners and a non-contact cantilever PPP-NCHR 5M, permitted to analyze the surface topography of the disks.

The scan rate was 0.1 Hz, and each disk was analyzed in four different areas of 10 µm × 10 µm, as previously described [29]. The Nanoscope Analysis software (1.5, Bruker, Milan, Itay) was used for measuring the average roughness, Ra, and the root-mean-square roughness of profile, Rq, with the respective standard deviations (SD).

#### 2.4.2. Fourier-Transform Infrared Spectroscopy

The X-ray diffraction (FTIR) analysis and light optical microscope survey were permitted to observe the morphology of the oxide layer of the samples [30]. For the superficial disk microstructure observations, an Olympus/PMG3 optical microscope (Olympus Corporation, Tokyo, Japan) was used.

The area of the disks covered by different colors was recorded by a Sony α330 digital camera (Sony, Konan, Minato-ku, Tokyo, Japan) and then, the NIS-Elements AR software (3.0, Nikon, Minato, Japan) performed the morphometric analysis. The measured parameters were expressed in percentage (Mean ± Standard Deviation).

#### 2.4.3. Scanning Electron Microscopy

The Scanning Electron Microscopy observation, SEM (JSM-6480LV; Jeol, Tokyo, Japan), was used to obtain the surface topography analysis using the instrument’s software, as previously described [31].

### 2.5. Statistical Analysis

The data analysis was performed by GraphPad Prism 6 software package (GraphPad Software, Inc., San Diego, CA 92108, USA). The Shapiro–Wilks normality test was performed, and the roughness surface means were evaluated between the study groups by the Kruskal–Wallis test followed by the Dunn–Bonferroni Post Hoc analysis. The level of significance for the analysis was *p* ≤ 0.05.

## 3. Results

**Figure 2:**
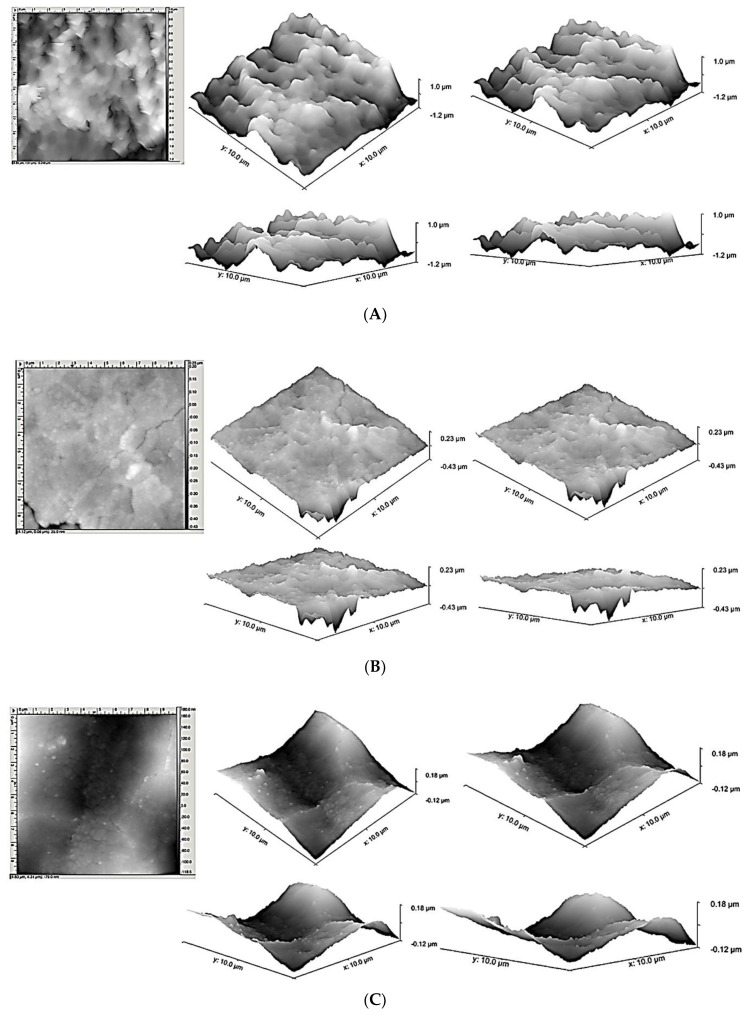
Atomic Force Microscope (AFM) images of (**A**) control, (**B**) the same samples after laser irradiation at 1 W, and (**C**) after laser irradiation at 4 W. Image A and B show a reduction of roughness surface.

**Figure 3:**
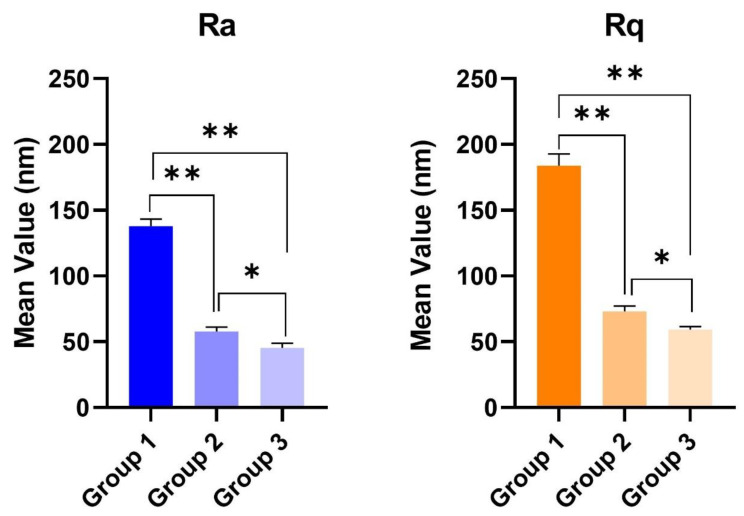
Surface roughness parameters of two differently treated titanium disks. Ra and Rq of Group 1 (no treatment) were significantly higher than Group 2 (1 W and 100 mJ/pulse) and Group 3 (4 W and 400 mJ/pulse) (** *p* < 0.01). A statistical difference was present between Groups 2 and 3 (* *p* < 0.05).

The AFM measured the roughness parameters in each of the ten disks and the mean values (±standard deviations) of average roughness (Ra) and root mean square roughness of profile (Rq) were calculated (Figure 2A–C). The field of view was also sufficient to detect the longer-range roughness due to sandblasting. The average Ra (±standard deviations) were 137.71 (±5.317), 57.83 (±3.136), and 45.21 (±3.461) nm for Groups 1, 2, and 3, respectively. The mean Rq (±standard deviations) were 183.92 (±8.391), 72.92 (±4.297), and 59.3 (±2.313) for Groups 1, 2, and 3, respectively. A significant statistical difference was detected for both roughness parameters between controls vs. Group 2 and 3, *p* < 0.05. A statistical difference (*p* < 0.05) was also present between Groups 2 and 3 (Table 1 and Figure 3).

**Figure 4:**
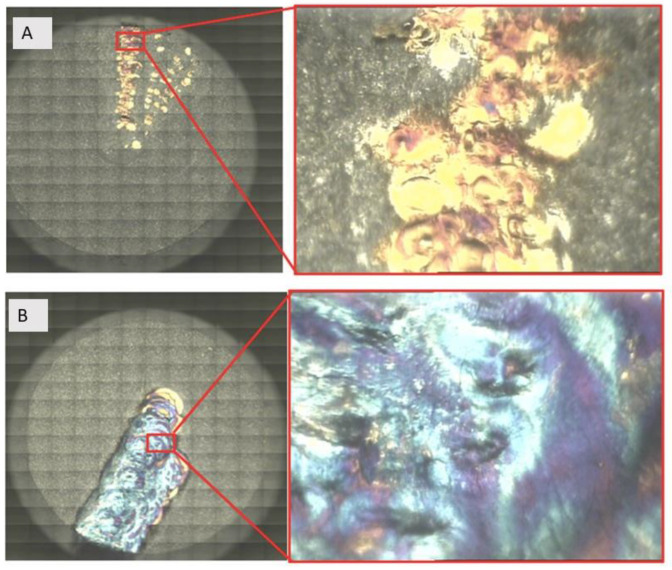
(**A**) The samples after laser irradiation at 1 W, and (**B**) after laser irradiation at 4 W. The untreated area and treated area show a different color distribution.

**Figure 5:**
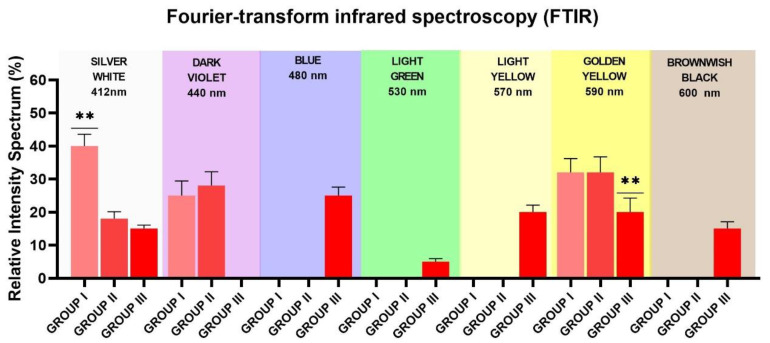
Fourier-transform infrared spectroscopy (FTIR) relative intensity spectrum bands of control and two differently treated titanium disks. Group 1 (no treatment), Group 2 (1 W and 100 mJ/pulse) and Group 3 (4 W and 400 mJ/pulse) (** = *p* < 0.01).

The achieved colors by means of FTIR followed a chromatic scale that went from silver-white to golden-yellow, to blue to light yellow, and then to light green and brownish-black (Figure 4A,B). In the control, Group 1, the color on the surface of the specimen was silver-white (40%), golden-yellow (32% ± 4.2%), and dark violet (25% ± 4.4%), and no microcracks were observed. In the test Group 2, the color on the surface of the specimen was silver-white (18% ± 2.2%), golden-yellow (32% ± 4.2%), and dark violet (28% ± 4.4%), and no microcracks were observed. In the test Group 3, the color on the surface of the specimen was silver-white (15% ± 1.2%), light-yellow (20% ± 2.1%), golden-yellow (20% ± 4.1%), blue (25% ± 2.5%), light-green (5% ± 0.9%), and then brownish-black (15% ± 1.9%). In the test Group 3, it was also possible to observe many microcracks. Such color changes on the samples were closely related to colored titanium oxides in the oxide layer. From the FTIR analysis, we acquired qualitative information about changes in the thickness of the oxide layer as the power laser irradiation. In all groups, the spectroscopic analysis by FTIR showed that absorption in the 400–700 cm^−1^ range changed with power laser irradiation, while the transmittance was increased after the laser irradiation in the Groups 2 and 3 (Table 2 and Figure 5). According to the described band, the disk surfaces were covered by different thickness of an amorphous TiO_2_ layer.

**Figure 6:**
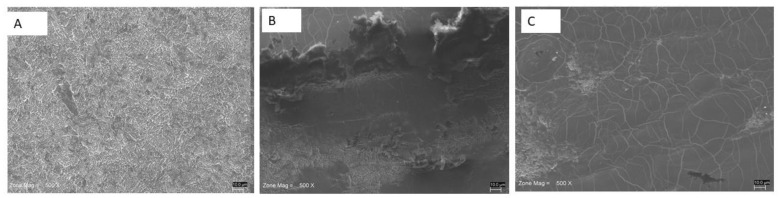
Scanning Electron Microscopy (SEM) analysis. (**A**) The disk surface of controls shows a nanoporous network structure of typical microroughness imparted by SLA (no treatment). (**B**) Group 2 showed delamination and deformation of the surface present in the laser irradiated areas. (**C**) Group 3 showed signs of coagulation, melting, and microfractures.

Scanning Electron Microscopy (SEM) analysis revealed a nanoporous network structure on the surface of the titanium disk of Group 1 (Figure 6A). The topography of the disk showed the typical microroughness imparted by SLA. Delamination and deformation of the surface were present in the laser irradiated areas. In particular, a loss of porosity, because of the extensive melting, and a relatively smooth surface were found in the irradiated disks. In particular, the titanium disks of Group 2 showed surface alterations, like exfoliation, melting of the material, and microcracks (Figure 6B). In Group 3, signs of coagulation, melting, and microfractures were observed (Figure 6A).

## 4. Discussion

The impact of two different protocols of Er: YAG laser irradiation on SLA titanium disks for the treatment of peri-implantitis has been evaluated in this study. The treatment of peri-implantitis includes granulation tissue removal, the decontamination of exposed implant surfaces, the application of antibacterial agent, and the smoothening of rough implant surfaces, thus rendering those surfaces less attractive for bacterial accumulation [32,33]. Hauser-Gerspachave et al. showed that bactericidal effects of Er: YAG increase with laser dose, but with the risk to surface alterations [15].

Consequently, it is essential to verify and quantify the impact of such therapies on the implant surface, in order to find the safer protocol. Indeed, nano-, micro-, and macro-morphology of titanium are essential for achieving and maintaining osseointegration and stable peri-implant soft tissue conditions. The surface properties have the potential to influence the osteoblast cell proliferation, differentiation, and morphology, and the expression of extracellular matrix components, integrins, and growth factors [34,35]. The outcomes presented here show that Er: YAG laser irradiation on SLA titanium disks can lead to the reduction of the roughness surface with modification of the oxide layer.

A variation of the superficial roughness has the potential to also modify the bacterial adhesion to the implant: it has been shown that by increasing mean Ra above 0.200 µm, the retention of plaque increases [36]. On the contrary, the presence of nano-roughness on the surface of titanium could exert an antibacterial activity, and some authors proposed a laser nano-texturing process of titanium in order to decrease the biofilm formation [37,38].

In this study, the surface modifications provided by the laser treatment were also visible macroscopically, with the color changing on the samples, that reached a dye that was near to that of the titanium oxides layer. These findings, supplemented by FTIR results, confirmed that our treatment enhanced the deposition of a new oxide layer mainly composed of amorphous TiO_2_. Moreover, the color of the disks changed significantly with the increase of power laser irradiation. It is well known that titanium is covered in the external layer by titanium oxides, like TiO_2_, TiO, and Ti_2_O_3_ [39]. Our findings suggest that laser irradiation increased the oxide layer of the TiO_2_ and TiO. TiO_3_ is dark-violet while TiO_2_ and TiO are respectively white- and golden-tinted [40]. However, the interpretation of the color change on titanium alloys is difficult. The presence and the quantification of the layer of oxidation is very important because it affects the wettability of the implant and consequently, the protein adsorption, the cellular adhesion and spreading, and the ability to promote differentiation of osteoblasts [41,42].

Recently, Ercan highlighted that the distance at which irradiation affects the power density is the principal parameter that influences the surface changes during laser treatment [43]. The laser irradiation of machined Ti at a power setting above 2 W resulted in the increased proportion of oxygen and decreased Ti, due to oxidation, with respect non irradiated disks; on the contrary, anodized titanium irradiated with the same settings resulted in the reduced proportion of O and increased Ti [24].

However, laser settings are also fundamental for the interaction with ti surfaces: the percentage of O in the superficial composition of machined Ti increased from about 2.5% in non-irradiated samples to 5.8%, 12.26%, 24.2%, and 35.0% after Er: YAG irradiation at 2, 3, 4, and 5 W, respectively [24].

Another interesting outcome of the present study is that there was a change in the nano-roughness surface of titanium grade 4. The roughness parameters decreased with the increase of laser settings in acid-etched (SLA) surfaces, and there was also a higher presence of cracks that developed after increasing irradiation at 400 mJ/10 Hz. These results are in accordance with another study [26]. The roughness of titanium is crucial because it is positively correlated to cellular adhesion, functional alterations, and proliferation of both osteoblastic cells and bacteria [34,35,44]. Galli et al. have shown that Er: YAG laser irradiation, 10 s at 200 mj/pulse and 10 Hz, promoted the formation of a melted area on SLA titanium, with the collapse of thin crest to flat, smooth plates [45]. These surface alterations were able to slow down the cellular proliferation, but did not alter the cellular differentiation. On the contrary, Wakim et al. have found no significant alteration on SLA titanium surfaces after multiple Er: YAG treatments with irradiation energy 50 mJ, 30 Hz frequency, 1.5 W power output, and 3.76 J/cm^2^ energy density, but in this protocol, a super short pulse of 50 us was applied [46]. As a result, reduction to surface micro-roughness could delay or prevent bone bacterial adhesion. The results of the present research indicated that laser irradiation also changes oxide layers, especially the TiO_2_. This change is significant because it reduces the number of bacteria on the titanium and produces more healthy peri-implant tissues [47].

This study further confirms that the choice of laser parameters, like the power settings, the presence of water cooling, the distance, the inclination, the type of movements of the tip during irradiation, and the time of light exposure, should be made with extreme attention, considering approved and tested protocols, in order not to damage the titanium surface.

The limitations of this study are the absence of cellular and microbiological testing. This is only a preliminary study that aims to evaluate the topographical alterations on SLA titanium disks after Er: YAG irradiations, but the biological consequence of the different protocols should be tested in further in vitro and in vivo experimentations. Moreover, in vivo, the presence of saliva and pH conditions could interfere with the chemical composition of titanium. Another limitation is that the surface roughness statistical analysis performed in this study comprehends only data on the vertical dimension of surface architecture and it should be enhanced by adding other roughness parameters that would allow to describe the texture morphology in terms of height/peaks distribution.

## 5. Conclusions

Both tested protocols of Er: YAG laser irradiation of SLA titanium provided some modifications to the topography of the surface of the samples.

However, the treatment at 4 W and 400 mJ/pulse could weaken the titanium surface, as shown by the presence of microcracks and signs of coagulation, melting, and microfractures.

## Figures and Tables

**Table 1:** Atomic Force Microscope (AFM) evaluation of the grade 4 titanium disks’ surface (n = 10 samples per group). Ra: Arithmetical mean deviation; Rq: Root mean square deviation (mean, standard deviation).

Samples’ Surface Roughness	Ra (nm)	Rq (nm)
Group 1	137.71 ± 5.317	183.92 ± 8.391
Group 2	57.83 ± 3.136	72.92 ± 4.297
Group 3	45.21 ± 3.461	59.3 ± 2.313

**Table 2:** Fourier-transform infrared spectroscopy (FTIR) evaluation of the grade 4 titanium disks’ surface: relative intensity spectrum bands of the study groups (mean, standard deviation). n.d. = not detectable.

Relative Intensity Spectrum Bands	Silver-White	Dark-Violet	Blue	Light-Green	Light-Yellow	Golden-Yellow	Brownwish-Black
Group 1	40% ± 3.6%	25% ± 4.4%	n.d.	n.d.	n.d.	32% ± 4.2%	n.d.
Group 2	18% ± 2.2%	28% ± 4.2%	n.d.	n.d.	n.d.	32% ± 4.4%	n.d.
Group 3	15% ± 1.2%	n.d.	25% ± 2.5%	5% ± 0.9%	20% ± 2.1%	20% ± 4.1%	15% ± 1.9%

## References

[1] Wang W.C., Lagoudis M., Yeh C.-W., Paranhos K.S. (2017). Management of peri-implantitis—A contemporary synopsis. Singap. Dent. J..

[2] Schwarz F., Derks J., Monje A., Wang H.-L. (2018). Peri-implantitis. J. Clin. Periodontol..

[3] Lafaurie G.I., Sabogal M.A., Castillo D.M., Rincón M.V., Gómez L.A., Lesmes Y.A., Chambrone L. (2017). Microbiome and Microbial Biofilm Profiles of Peri-Implantitis: A Systematic Review. J. Periodontol..

[4] Ballini A., Cantore S., Farronato D., Cirulli N., Inchingolo F., Papa F., Malcangi G., Inchingolo A.D., Dipalma G., Sardaro N. (2015). Periodontal disease and bone pathogenesis: The crosstalk between cytokines and porphyromonas gingivalis. J. Biol. Regul. Homeost. Agents.

[5] Berglundh T., Armitage G., Araujo M.G., Avila-Ortiz G., Blanco J., Camargo P.M., Chen S., Cochran D., Derks J., Figuero E. (2018). Peri-implant diseases and conditions: Consensus report of workgroup 4 of the 2017 World Workshop on the Classification of Periodontal and Peri-Implant Diseases and Conditions. J. Periodontol..

[6] Renvert S., Polyzois I. (2018). Treatment of pathologic peri-implant pockets. Periodontology 2000.

[7] Novaes Junior A.B., Ramos U.D., Rabelo M.d.S., Figueredo G.B. (2019). New strategies and developments for peri-implant disease. Braz. Oral Res..

[8] Daubert D.M., Weinstein B.F. (2019). Biofilm as a risk factor in implant treatment. Periodontology 2000.

[9] Radunović M., Petrini M., Vlajic T., Iezzi G., Di Lodovico S., Piattelli A., D’Ercole S. (2020). Effects of a novel gel containing 5-aminolevulinic acid and red LED against bacteria involved in peri-implantitis and other oral infections. J. Photochem. Photobiol. B Biol..

[10] Petrini M., Spoto G., Scarano A., D’Arcangelo C., Tripodi D., Di Fermo P., D’Ercole S. (2019). Near-infrared LEDS provide persistent and increasing protection against *E. faecalis*. J. Photochem. Photobiol. B.

[11] D’Ercole S., Spoto G., Trentini P., Tripodi D., Petrini M. (2016). In vitro inactivation of Enterococcus faecalis with a led device. J. Photochem. Photobiol. B Biol..

[12] Petrini M., Trentini P., Tripodi D., Spoto G., D’Ercole S. (2017). In vitro antimicrobial activity of LED irradiation on Pseudomonas aeruginosa. J. Photochem. Photobiol. B Biol..

[13] Bush K., Courvalin P., Dantas G., Davies J., Eisenstein B., Huovinen P., Jacoby G.A., Al E. (2011). Tackling antibiotic resistance. Nat. Rev. Microbiol..

[14] Scarano A., Nardi G., Murmura G., Rapani M., Mortellaro C. (2016). Evaluation of the Removal Bacteria on Failed Titanium Implants after Irradiation with Erbium-Doped Yttrium Aluminium Garnet Laser. J. Craniofac. Surg..

[15] Hauser-Gerspach I., Mauth C., Waltimo T., Meyer J., Stübinger S. (2014). Effects of Er:YAG laser on bacteria associated with titanium surfaces and cellular response in vitro. Lasers Med. Sci..

[16] Takagi T., Aoki A., Ichinose S., Taniguchi Y., Tachikawa N., Shinoki T., Meinzer W., Sculean A., Izumi Y. (2018). Effective removal of calcified deposits on microstructured titanium fixture surfaces of dental implants with erbium lasers. J. Periodontol..

[17] Schwarz F., John G., Schmucker A., Sahm N., Becker J. (2017). Combined surgical therapy of advanced peri-implantitis evaluating two methods of surface decontamination: A 7-year follow-up observation. J. Clin. Periodontol..

[18] Tosun E., Tasar F., Strauss R., Kıvanc D.G., Ungor C. (2012). Comparative Evaluation of Antimicrobial Effects of Er:YAG, Diode, and CO_2_ Lasers on Titanium Discs: An Experimental Study. J. Oral Maxillofac. Surg..

[19] Romeo U., Libotte F., Palaia G., Del Vecchio A., Tenore G., Visca P., Nammour S., Polimeni A. (2012). Histological in vitro evaluation of the effects of Er:YAG laser on oral soft tissues. Lasers Med. Sci..

[20] Lide D.R. (2009). CRC Handbook of Chemistry and Physics.

[21] Sculean A., Schwarz F., Becker J. (2005). Anti-infective therapy with an Er:YAG laser: Influence on peri-implant healing. Expert Rev. Med. Devices.

[22] Diamanti M.V., Pedeferri M.P. (2007). Effect of anodic oxidation parameters on the titanium oxides formation. Corros. Sci..

[23] Schwarz F., Sculean A., Berakdar M., Szathmari L., Georg T., Becker J. (2003). In vivo and in vitro effects of an Er:YAG laser, a GaAlAs diode laser, and scaling and root planing on periodontally diseased root surfaces: A comparative histologic study. Lasers Surg. Med..

[24] Varshney D., Dodiya N. (2013). Electrical resistivity of alkali metal doped manganites LaxAyMnwO3 (A = Na, K, Rb): Role of electron–phonon, electron–electron and electron–magnon interactions. Curr. Appl. Phys..

[25] Ayobian-Markazi N., Karimi M., Safar-Hajhosseini A. (2015). Effects of Er: YAG laser irradiation on wettability, surface roughness, and biocompatibility of SLA titanium surfaces: An in vitro study. Lasers Med. Sci..

[26] Stubinger S., Etter C., Miskiewicz M., Homann F., Saldamli B., Wieland M., Sader R. (2010). Surface alterations of polished and sandblasted and acid-etched titanium implants after Er:YAG, carbon dioxide, and diode laser irradiation. Int. J. Oral Maxillofac. Implants.

[27] Kreisler M., Götz H., Duschner H. (2002). Effect of Nd:YAG, Ho:YAG, Er:YAG, CO_2_, and GaAIAs laser irradiation on surface properties of endosseous dental implants. Int. J. Oral Maxillofac. Implants.

[28] Kim H.-K., Park S.-Y., Son K., Kim Y.-G., Yu W.-J., Lee K.-B., Lee J.-M. (2020). Alterations in Surface Roughness and Chemical Characteristics of Sandblasted and Acid-Etched Titanium Implants after Irradiation with Different Diode Lasers. Appl. Sci..

[29] Scarano A., Lorusso F., Orsini T., Morra M., Iviglia G., Valbonetti L. (2019). Biomimetic Surfaces Coated with Covalently Immobilized Collagen Type I: An X-Ray Photoelectron Spectroscopy, Atomic Force Microscopy, Micro-CT and Histomorphometrical Study in Rabbits. Int. J. Mol. Sci..

[30] Ettorre V., De Marco P., Zara S., Perrotti V., Scarano A., Di Crescenzo A., Petrini M., Hadad C., Bosco D., Zavan B. (2016). In vitro and in vivo characterization of graphene oxide coated porcine bone granules. Carbon N. Y..

[31] Scarano A., Petrini M., Mastrangelo F., Noumbissi S., Lorusso F. (2020). The Effects of Liquid Disinfection and Heat Sterilization Processes on Implant Drill Roughness: Energy Dispersion X-ray Microanalysis and Infrared Thermography. J. Clin. Med..

[32] Carinci F., Lauritano D., Bignozzi C.A., Pazzi D., Candotto V., De Oliveira P.S., Scarano A., De Oliveira S. (2019). A New Strategy Against Peri-Implantitis: Antibacterial Internal Coating. Int. J. Mol. Sci..

[33] Romeo E., Ghisolfi M., Murgolo N., Chiapasco M., Lops D., Vogel G. (2004). Therapy of peri-implantitis with resective surgery. Clin. Oral Implants Res..

[34] Schwarz F., Wieland M., Schwartz Z., Zhao G., Rupp F., Geis-Gerstorfer J., Schedle A., Broggini N., Bornstein M.M., Buser D. (2009). Potential of chemically modified hydrophilic surface characteristics to support tissue integration of titanium dental implants. J. Biomed. Mater. Res. Part B Appl. Biomater..

[35] Romanos G., Crespi R., Barone A., Covani U. (2010). Osteoblast attachment on titanium disks after laser irradiation. Int. J. Oral Maxillofac. Implants.

[36] Bollen C.M., Lambrechts P., Quirynen M. (1997). Comparison of surface roughness of oral hard materials to the threshold surface roughness for bacterial plaque retention: A review of the literature. Dent. Mater..

[37] Amoroso P.F., Adams R.J., Waters M.G.J., Williams D.W. (2006). Titanium surface modification and its effect on the adherence of Porphyromonas gingivalis: An in vitro study. Clin. Oral Implants Res..

[38] Ionescu A.C., Brambilla E., Azzola F., Ottobelli M., Pellegrini G., Francetti L.A. (2018). Laser microtextured titanium implant surfaces reduce in vitro and in situ oral biofilm formation. PLoS ONE.

[39] Scarano A., Crocetta E., Quaranta A., Lorusso F. (2018). Influence of the Thermal Treatment to Address a Better Osseointegration of Ti6Al4V Dental Implants: Histological and Histomorphometrical Study in a Rabbit Model. Biomed Res. Int..

[40] Peng W., Zeng W., Zhang Y., Shi C., Quan B., Wu J. (2013). The Effect of Colored Titanium Oxides on the Color Change on the Surface of Ti-5Al-5Mo-5V-1Cr-1Fe Alloy. J. Mater. Eng. Perform..

[41] Zhu X., Chen J., Scheideler L., Reichl R., Geis-Gerstorfer J. (2004). Effects of topography and composition of titanium surface oxides on osteoblast responses. Biomaterials.

[42] van Kooten T.G., Schakenraad J.M., van der Mei H.C., Busscher H.J. (1992). Influence of substratum wettability on the strength of adhesion of human fibroblasts. Biomaterials.

[43] Ercan E., Candirli C., Arin T., Kara L., Uysal C. (2015). The effect of Er,Cr:YSGG laser irradiation on titanium discs with microtextured surface morphology. Lasers Med. Sci..

[44] Stolzoff M., Burns J.E., Tobin E.J., Nguyen C., De La Torre N., Golshan N.H., Ziemer K.S., Webster T.J. (2017). Decreased bacterial growth on titanium nanoscale topographies created by ion beam assisted evaporation. Int. J. Nanomed..

[45] Galli C., Macaluso G.M., Elezi E., Ravanetti F., Cacchioli A., Gualini G., Passeri G. (2011). The Effects of Er:YAG Laser Treatment on Titanium Surface Profile and Osteoblastic Cell Activity: An In Vitro Study. J. Periodontol..

[46] Nejem Wakim R., Namour M., Nguyen H., Peremans A., Zeinoun T., Vanheusden A., Rompen E., Nammour S. (2018). Decontamination of Dental Implant Surfaces by the Er:YAG Laser Beam: A Comparative in Vitro Study of Various Protocols. Dent. J..

[47] Scarano A., Piattelli A., Polimeni A., Di Iorio D., Carinci F. (2010). Bacterial Adhesion on Commercially Pure Titanium and Anatase-Coated Titanium Healing Screws: An In Vivo Human Study. J. Periodontol..

